# Dimerization of propargyl and homopropargyl 6-azido-6-deoxy-glycosides upon 1,3-dipolar cycloaddition

**DOI:** 10.3762/bjoc.4.30

**Published:** 2008-08-13

**Authors:** Nikolas Pietrzik, Daniel Schmollinger, Thomas Ziegler

**Affiliations:** 1 Institute of Organic Chemistry, University of Tuebingen, Auf der Morgenstelle 18, 72076 Tuebingen, Germany.

**Keywords:** click reaction, cyclodimerization, glycosides, triazoles

## Abstract

Copper-catalyzed, thermal or microwave promoted 1,3-dipolar cycloaddition (Click Reaction) of 2-propynyl and 3-butynyl 2,3,4-tri-*O*-acetyl-6-azido-6-deoxy-glycopyranosides in the D-gluco, D-galacto and D-manno series afford the corresponding dimeric cycloaddition products.

## Introduction

**Figure 1:**
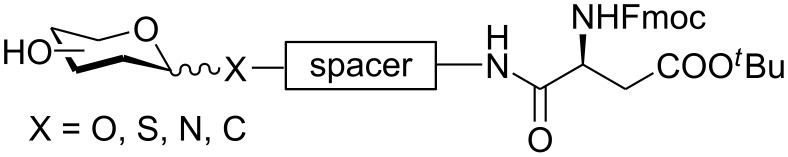
Schematic representation of glycosylated building blocks for the combinatorial synthesis of glycopeptides.

Our ongoing interest in constructing combinatorial libraries of highly glycosylated beta-peptides that can mimic specific oligosaccharide-protein interactions prompted us to further search for efficient routes toward glycosylated amino acid building blocks derived from asparaginic acid in which the glycon is bound to C-1 of the asparaginic acid through variable spacers (Figure 1). Previously, we have prepared a series of glycosylated asparaginic acid building blocks containing as spacers either simple alkyl chains [1], or amino alcohols [2–3]. Such building blocks have been shown to be well suited for combinatorial solid phase or spot synthesis of libraries of highly glycosylated peptides, some members of which were indeed shown to behave like oligosaccharide mimics capable to specifically bind lectins [1,4].

In order to increase the structural diversity of the aforementioned building blocks, we contemplated using as the spacer entity 1,2,3-triazoles which are known to be easily generated through a copper-catalyzed 1,3-dipolar cycloaddition of an organic azide and an alkynyl derivative (Click Reaction) [5–7]. For review articles on copper-catalyzed Click Reactions see references [8–11]. Recently, we applied this approach to a series of 1,2,3-triazole containing per-*O*-acetyl-glycosides which were prepared by copper-catalyzed 1,3-dipolar cycloaddition either between fully acetylated propargyl 1-thio-glycosides and *t*-butyl (*S*)-4-azido-3-fluorenylmethyloxycarbamido-butyrate or between Fmoc-L-Asp(O*^t^*Bu)-propargyl amide and 2,3,4,6-tetra-*O*-acetyl-glycosyl azides and ethyl 2,3,4-tri-*O*-acetyl-6-azido-6-deoxy-1-thio-glycosides, respectively [12]. In order to increase the structural diversity of glycosyl amino acid building blocks containing 1,2,3-triazole spacers even more, we next looked at the possibility to use glycosides bearing both, azido and alkynyl groups in copper-catalyzed 1,3-cycloadditions. The results are presented here.

## Results and Discussion

**Scheme 1:**
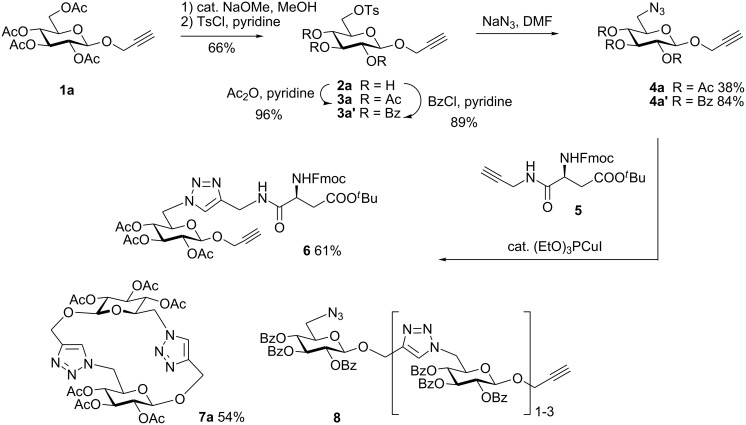
Synthesis and reaction of compounds **4a** and **4a**'.

First, 2-propynyl 6-azido-6-deoxy-2,3,4-tri-*O*-acetyl-β-D-glucopyranoside (**4a**) was prepared by the following sequence. 2-Propynyl 2,3,4,6-tetra-*O*-acetyl-β-D-glucopyranoside (**1a**) [13] was Zemplén-deacetylated with a catalytic amount of sodium methanolate in methanol. Next, thus obtained crude 2-propynyl β-D-glucopyranoside was regioselectively tosylated at position 6 [14] followed by chromatographic purification to afford 6-*O*-*p*-tolylsulfonyl-glucoside **2a** in 66% yield. Acetylation of the latter with acetic anhydride in pyridine gave crude tri-*O*-acetyl-6-*O*-*p*-tolylsulfonyl-glucoside **3a** which was sufficiently pure for the next step. Treatment of **3a** with NaN_3_ in DMF finally afforded 6-azido-6-deoxy-glucoside **4a** in 38% yield. When glucoside **4a** was reacted with asparaginic propargyl amide derivative **5** [12] in the presence of (EtO)_3_PCuI as catalyst and with or without microwave irradiation [15], the induced 1,3-dipolar cycloaddition between the alkynyl and azide moieties (Click Reaction) afforded compound **6** in variable medium yields of approximately 60%. The yield depended on the reaction conditions under which the cycloaddition was carried out. Several byproducts were formed during this cycloaddition reaction which, however, could not be separated and characterized. The amount of these byproducts increased at higher reaction temperatures or upon irradiation with microwave. It was anticipated that the byproducts which lowered the yield of compound **6** might be decomposition products of the starting material **4a**. Therefore, the more stable benzoylated glucoside **3a'** was prepared from **2a**, and converted into the azide **4a'** in 89% and 84% yield, respectively. Treatment of **4a'** with **5** under Cu(I)-catalysis, however, only resulted in a complex mixture of reaction products from which no uniform product could be isolated. Therefore, it was concluded that **4a** and **4a'** may have reacted with themselves resulting in products of oligomerization. Indeed, when **4a** was treated with a catalytic amount of (EtO)_3_PCuI , TLC (ethyl acetate/*n*-hexane 1:1) revealed the formation of one faster moving product along with a complex mixture of slower moving products with mobility similar to those previously observed. Careful inspection of the products revealed that dimerisation of **4a** occurred, affording the dimeric glycoside **7a** beside products of oligomerization (Scheme 1). The reaction proceeded significantly slower than the coupling of **4a** and **5**. A faster reaction occurred upon irradiation with microwave, which also gave a higher yield (54%) of **7a**. Benzoylated glycoside **4a'** did not give any product of dimerization though. Only oligomers **8** were observed in this case (for details see Supporting Information File 1).

At first, it was unclear whether **7a** was formed by an intramolecular cyclization or a dimerization of **4a** since its concentration-dependent ESI-MS and MALDI-TOF-MS spectra both showed peaks corresponding to the molecular mass of **4a** and **7a**, respectively. However, the dimeric structure of compound **7a** was finally unambiguously assigned by NMR spectroscopy and field desorption (FD) mass spectrometry. The NMR spectra of **7a** showed no conformative anomalies of the pyranose ring what would have been expected if **7a** would have been the product of intramolecular 1,3-dipolar cycloaddition of the azido group and the 2-propynyl aglycon in the starting material **4a**.

The oligomerization of glycosides containing both, an azido and an alkynyl group upon copper-catalyzed Click-Reaction had been observed previously in two instances. Gin and coworker recently found that 2,3,6-tri-*O*-benzyl-4-*O*-(2-propynyl)-α-D-mannopyranosyl azide affords a cyclic trimer upon 1,3-dipolar cycloaddition of its azido moiety to its propynyl moiety while the corresponding α-1,4-linked manno-disaccharide afforded a cyclic dimer similar to compound **7a** [16]. Jarosz et al. also recently reported about the copper catalyzed reaction of 6-azido-1',2,3,3',4,4'-hexa-*O*-benzyl-6-deoxy-6'-propargyl-sucrose to afford either a product of intramolecular cyclization or a dimeric product, depending on the reaction conditions [17]. Likewise, Vasella reported the thermal intramolecular 1,3-dipolar cycloaddition of protected 2-azidoethyl 4^5^-*O*-(2-propynyl)-malto-hexaoside, giving the corresponding isomeric macrocyclic derivatives [18]. In the light of Gin's and Jarosz's results and our own unexpected finding that **4a** can form cyclic dimers upon copper-catalyzed Click-Reaction, we investigated several other 2-propynyl and 3-butynyl 6-azido-6-deoxy-glycosides **4** in order to probe their ability to form similar cyclic dimers **7**.

**Scheme 2:**
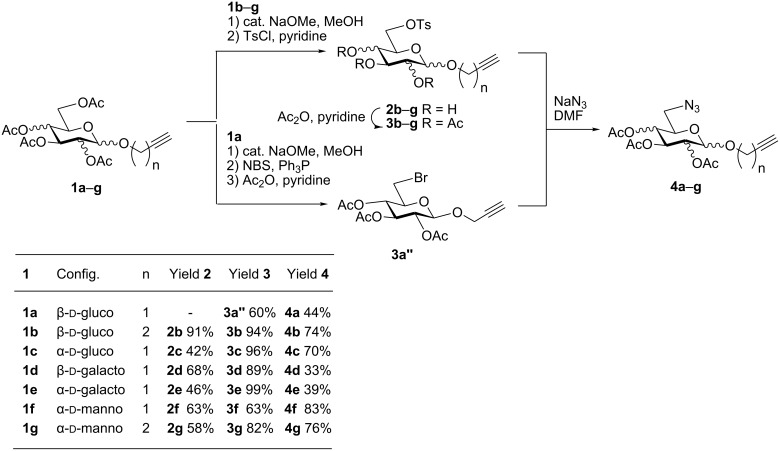
Preparation of compounds **4a**–**g**.

First, an alternative route to 2-propynyl 6-azido-6-deoxy-glucoside **4a** was attempted (Scheme 2). Compound **1a** was deacetylated and treated with *N*-bromosuccinimide and triphenylphosphine in DMF according to Hanessian's procedure [19] followed by reacetylation of the OH-groups with acetic anhydride in pyridine to afford 2-propynyl 6-bromo-6-deoxy-2,3,4-tri-*O*-acetyl-β-D-glucopyranoside (**3a''**) in 60% yield. Next, the latter was stirred with NaN_3_ in DMF (48 h, 65 °C) to afford **4a** in 44% yield. The preparation of compound **4a** *via* the corresponding tosylate **3a** was somewhat more convenient than the synthesis *via* the 6-bromo-6-deoxy counterpart **3a''** and resulted in a similar overall yield. Therefore, all other 6-azido-6-deoxy-glycosides **4** were prepared via the corresponding tosylates **3** as described above. Scheme 2 summarizes the yields for the preparation of the tosylates **3** and 6-azido-6-deoxy-glycosides **4**. Starting materials **1** were prepared following known procedures for **1a** [13,20], **1b** [21], **1d** [13], **1f** [20,22] and **1g** [21]. 2-Propynyl 2,3,4,6-tetra-*O*-acetyl-α-D-glycopyranosides **1c** and **1e** have not been described previously. They were prepared from D-glucose and D-galactose in 20% and 22% yield, respectively via classical Fischer-Glycosylation in 2-propynol as the solvent under acidic conditions followed by acetylation of the intermediate glycosides and chromatographic separation of the anomeric acetates.

Next, glycosides **4a**–**g** were submitted to dimerization by 1,3-dipolar cycloaddition reaction. As the catalyst, 10 mol% (EtO)_3_PCuI was applied and used along with three equivalents diisopropyl ethylamine in toluene [15]. Microwave irradiation [23] reduced the reaction time significantly but also resulted in decomposition of the starting material in some cases. Table 1 summarizes the results for the dimerization of **4a**–**g** to **7a**–**g**.

**Table 1:** Dimerization of Glycosides **4a**–**g** under Cu-Catalysis.

Entry	Glycoside **4**	Product **7**	Conditions	Yield

1	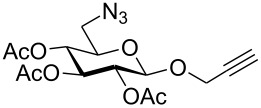 **4a**	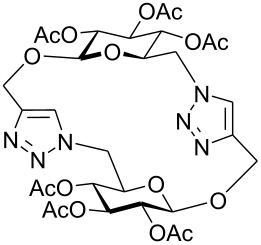 **7a**	12 h rt 1 h 80 °C, 20 W MW	54% 20%
2	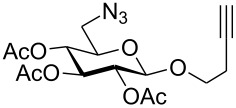 **4b**	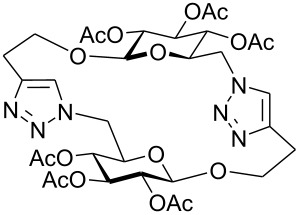 **7b**	12 h rt 1 h 80 °C, 20 W MW	- 32%
3	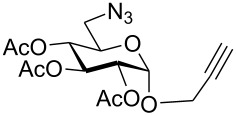 **4c**	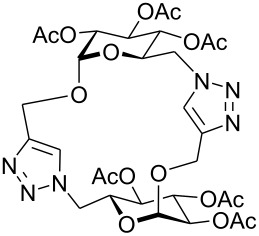 **7c**	12 h rt 1 h 80 °C, 20 W MW	14% -
4	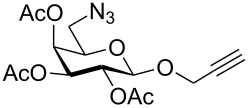 **4d**	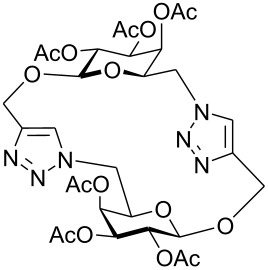 **7d**	12 h rt 1 h 80 °C, 20 W MW	28% -
5	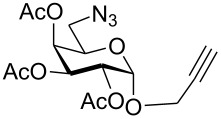 **4e**	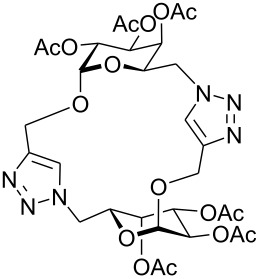 **7e**	12 h rt 1 h 80 °C, 20 W MW	- -
6	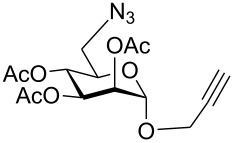 **4f**	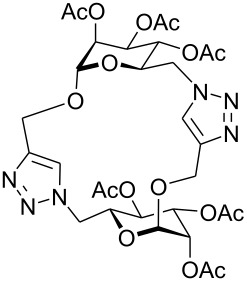 **7f**	12 h rt 1 h 80 °C, 20 W MW	- 30%
7	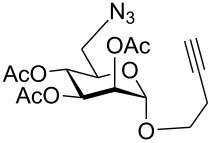 **4g**	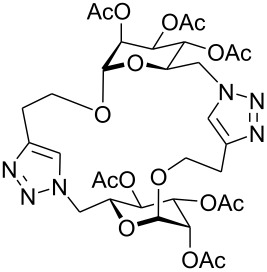 **7g**	12 h rt 1 h 80 °C, 20 W MW	- 53%

Yields of the copper-catalyzed dimerizations were low to medium (14–54%) and depended on the sugar moiety, the anomeric configuration and the ring size which was formed during the Click-Reaction. In general, no other cyclization product could be isolated from the reaction mixtures although significant amounts of byproducts were formed. These byproducts were slower moving compounds on TLC (ethyl acetate/*n*-hexane 1:1) and appeared to be products of oligomerization of the starting material **4**. In the case of benzoylated glycoside **4a'** where no cyclic dimer could be isolated from the complex reaction mixture, FAB MS of the purified mixture indeed revealed the presence of linear dimers, trimers and tetramers.

α-Galactoside **4e** did not give any isolable dimer **7e** at all (*cf.* Table 1, entry 5). Similarly, α-glucoside **4c** resulted in a lower yield of the corresponding dimer compared to β-glucoside **4a** (*cf.* Table 1, entries 1 and 3). This may be attributed to a significant ring-strain in the α-linked dimers. For example, the ^1^H NMR of compound **7c** showed an unusually small coupling constant between H-1 and H-2 (<1.0 Hz) and H-2 and H-3 (3.1 Hz) which is indicative that the sugar moieties in **7c** are no longer in a chair conformation (see Table 1 in the Supporting Information File 1). No such effects were observed in the manno series though (*cf.* Table 1, entries 6 and 7). Here, the corresponding dimers **7f** and **7g** showed regular coupling constants in their NMR spectra.

The effect of microwave irradiation on the outcome of the dimerization is somewhat confusing. In general, microwave irradiation resulted in a faster reaction, *i.e.* faster disappearance of the starting material (*cf.* Table 1, entry 1). Similar accelerations of Click-Reactions upon microwave irradiation had been observed previously as well [15]. However, the higher temperature associated with the microwave irradiation also resulted in a more pronounced decomposition of the starting material, and thus resulted in a lower yield of the dimers (*cf.* Table 1, entries 1, 3 and 4) while heating of the reaction mixture alone resulted in complex product mixtures from which no dimerization products could be isolated. In the case of compounds **4b** and **4e**–**g**, no reaction occurred at room temperature (*cf.* Table 1, entries 2 and 5–7).

## Conclusion

We describe for the first time the copper-catalyzed dimerization of simple acetylated 2-propynyl and 3-butynyl 6-azido-6-deoxy-glycosides in the gluco, galacto and manno series leading to macrocyclic rings containing two sugar moieties and two 1,2,3-triazole moieties. For instance, such compounds may function as novel ligands for the preparation of metal complexes [24]. Further examples for cyclizations of other azido-alkynyl-glycosides are under investigation.

## Supporting Information

File 1 Experimental Data
