# Supplementary material for: Dimerization of propargyl and homopropargyl 6-azido-6-deoxy-glycosides upon 1,3-dipolar cycloaddition
Source: Beilstein J Org Chem. 2008 Aug 13;4:30. doi: 10.3762/bjoc.4.30 (PMC2533435; doi:10.3762/bjoc.4.30)
Supplement: File 1 — Experimental Data [file Beilstein_J_Org_Chem-04-30-s001.doc]

**Supporting Information**

Dimerization of propargyl and homopropargyl 6-azido-6-deoxy-glycosides upon 1,3-dipolar cycloaddition

Nikolas Pietrzik, Daniel Schmollinger and Thomas Ziegler*

Address: Institute of Organic Chemistry, University of Tuebingen, Auf der Morgenstelle 18, 72076 Tuebingen, Germany.

Email: Thomas Ziegler* - [thomas.ziegler@uni-tuebingen.de](mailto:thomas.ziegler@uni-tuebingen.de)

* Corresponding author

**Experimental Data**

**General**

All solvents were dried and distilled prior to their use. Reactions were performed under argon and monitored by TLC on Polygram Sil G/UV silica gel plates from Machery & Nagel. Detection was affected by charring with H_2_SO_4_ (5% in EtOH) or by inspection of the TLC plates under UV light. NMR spectra were recorded on a Bruker ARX 250 spectrometer at 100 MHz for proton spectra and 62.9 MHz for carbon spectra, and on a Bruker Avance 400 spectrometer at 400 MHz for proton spectra and 100 MHz for carbon spectra. Tetramethylsilane was used as the internal standard. NMR data are summarized in Tables 1 and 2. FAB MS was performed on a Finnigan MAT TSQ 70 spectrometer. HRFD MS was performed on a Bruker FT-ICR spectrometer. Elemental analyses were performed on a Hekatech Euro 3000 CHN analyzer. Optical rotations were measured with a Perkin-Elmer Polarimeter 341. Reactions under microwave irradiation were performed in a microwave reactor CEM Discover at 80 °C, 2.45 GHz and 20 W. Preparative chromatography was performed on silica gel (0.032-0.063 mm) from Machery & Nagel using different mixtures of solvents as eluent. Compounds **1a** [1,2], **1b** [3], **1d** [1], **1f** [2,4] and **1g** [3] were prepared according to the respective literature procedures.

**1c**

**2-Propynyl 2,3,4,6-tetra-*O*-acetyl-α-D-glucopyranoside (1c)**

Acetyl chloride (2 ml) was added to 2-propynol (100 ml) and the mixture was heated to 100 °C. Dry D-glucose (10 g, 55.56 mmol) was added in small portions to this mixture and stirring at 100 °C was continued for 4 h after the last addition of glucose. After cooling the mixture to room temperature, BaCO_3_ (5 g) was added with stirring and the slurry was filtered through a layer of Celite. The filtrate was concentrated in vacuo and the resulting oil was dissolved in pyridine (250 ml). The solution was cooled to 0 °C, acetic anhydride (83 ml, 870 mmol) was added, the mixture warmed to room temperature and stirred for 12 h. Ethanol (100 ml) was added and the mixture was concentrated in vacuo. Column chromatography of the residue (n-hexane/ethyl acetate 2:1) afforded **1c** (4.22 g, 20%). [α]_D_^20^ = +125.3 (*c* 1.0, CHCl_3_). FAB MS: m/z = 387.1 (M+H)^+^, 331.0 (M-OCH_2_CCH)^+^.

**1e**

**2-Propynyl 2,3,4,6-tetra-*O*-acetyl-α-D-galactopyranoside (1e)**

Treatment of D-galactose (10 g, 55.56 mmol) as described for the preparation of **1c** afforded **1e** (4.72 g, 22%). [α]_D_^20^ = +145.0 (*c* 1.0, CHCl_3_). FAB MS: m/z = 387.2 (M+H)^+^, 331.1 (M-OCH_2_CCH)^+^.

**2a**

**2-Propynyl 6-*O*-*p*-tolylsulfonyl-β-D-glucopyranoside (2a)**

To a solution of 2-propynyl 2,3,4,6-tetra-*O*-acetyl-β-D-glucopyranoside (**1a**) [1,2] (3.8 g, 10.0 mmol) in MeOH (30 ml) was added a catalytic amount of NaOMe (500 μl of a 1 M solution in MeOH). The solution was stirred at room temperature for 2 h, and neutralized by addition of 1 M aqueous HCl solution. Concentration in vacuo and co-evaporation with toluene afforded crude 2-propynyl β-D-glucopyranoside which was sufficiently pure for the next step. A solution of crude 2-propynyl β-D-glucopyranoside (1.1 g, 5.0 mmol) in pyridine (20 ml) was cooled to 0 °C and *p*-toluenesulfonyl chloride (1.5 g, 8.0 mmol) was added portionwise. The mixture was stirred for 6 h until TLC indicated complete consumption of the starting material. Water (50 ml) was added, the resulting solution saturated with NaCl and extracted with ethyl acetate (3 x 50 ml). The combined organic extracts were dried over Na_2_SO_4_, filtered and concentrated in vacuo. Column chromatography of the residue (MeOH/CHCl_3_ 1:3) afforded **2a** (1.23 g, 66%) as a slightly yellow oil which slowly decomposes upon storage at room temperature, and which should be used immediately for the next step. [α]_D_^20^ = -33.1 (*c* 1.0, MeOH).

**3a**

**2-Propynyl 2,3,4-tri-*O*-acetyl-6-*O*-*p*-tolylsulfonyl-β-D-glucopyranoside (3a)**

A solution of **2a** (0.93 g, 5.0 mmol) in pyridine (30 ml) was cooled to 0 °C, acetic anhydride (2.2 ml, 24.0 mmol) was added, and the solution was stirred at room temperature for 12 h. Ethanol (50 ml) was added, the mixture concentrated in vacuo, and traces of pyridine were removed by co-evaporation of toluene to give **3a** (2.4 g, 96%) which was used for the next step without further purification. FAB MS: m/z = 443.0 [M – OCH_2_CCH]^+^. An analytical sample was obtained by column chromatography (ethyl acetate/n-hexane 1 :1). [α]_D_^20^ = +40.3 (*c* 1, CHCl_3_). Anal. Calcd for C_22_H_26_O_11_S (498.5): C 53.01, H 5.26. Found: C 53.05, H 5.26.

**3a’**

**2-Propynyl 2,3,4-tri-*O*-benzoyl-6-*O*-*p*-tolylsulfonyl-β-D-glucopyranoside (3a’)**

A solution of **2a** (0.93 g, 5.0 mmol) in pyridine (30 ml) was cooled to 0 °C, benzoyl chloride (3.5 ml, 30.0 mmol) was added, and the solution was stirred at room temperature for 12 h. Ethanol (50 ml) was added, the mixture concentrated in vacuo, and traces of pyridine were removed by co-evaporation with toluene. Column chromatography (ethyl acetate/n-hexane 1:1) of the residue gave **3a’** (3.1 g, 89%). [α]_D_^20^ = + 13.5 (*c* 1, CHCl_3_). FAB MS: m/z = 629.2 [M – OCH_2_CCH]^+^. Anal. Calcd for C_37_H_32_O_11_S (684.7): C 64.90, H 4.71. Found: C 64.94, H 4.83.

**3a’’**

**2-Propynyl 2,3,4-tri-*O*-acetyl-6-bromo-6-deoxy-β-D-glucopyranoside (3a’’)**

Ph_3_P (4.8 g, 18.33 mmol) was added in small portions at 0 °C to a stirred solution of 2-propynyl β-D-glucopyranoside (2.0 g, 9.16 mmol), freshly prepared by deacetylation of **1a** (3.48 g, 9.16 mmol) as described for the preparation of **2a**, and NBS (3.27 g, 18.33 mmol) in DMF (100 ml). The mixture was heated to 50 °C and n-BuOH (50 ml) and MeOH (50 ml) were added. After cooling to room temperature, the mixture was concentrated in vacuo, the resulting syrup mixed with dichloromethane (20 ml) and extracted with water (3 x 75 ml). The combined aqueous extracts were neutralized with basic ion exchange resin and concentrated in vacuo. The residue was dissolved in pyridine (100 ml) and acetic anhydride (8.4 ml) was added under cooling with an ice bath. After stirring at room temperature for 12 h, the mixture was poured onto crushed ice and extracted with dichloromethane (3 x 50 ml). The combined organic extracts were washed with aqueous NaHCO_3_, dried over Na_2_SO_4_, filtered and concentrated. Recrystallisation of the residue from diethyl ether afforded **3a’’** (2.32 g, 60%). M.p. 114.2 °C. [α]_D_^20^ = -27.2 (*c* 1.0, CHCl_3_). FAB MS: m/z = 407.0. (M+H)^+^.

**4a**

**2-Propynyl 2,3,4-tri-*O*-acetyl-6-azido-6-deoxy-β-D-glucopyranoside (4a)**

A. A solution of **3a** (2.5 g, 5.0 mmol) and NaN_3_ (4.0 g, 60.0 mmol) in DMF/H_2_O (9:1, 55 ml) was heated to 80 °C for 48 h until TLC indicated complete consumption of the starting material. The solution was poured onto crushed ice and the mixture was extracted with dichloromethane (3 x 50 ml). The combined organic extracts were dried over Na_2_SO_4_, filtered and concentrated in vacuo. Column chromatography (n-hexane/ethyl actate 1:1) of the residue afforded **4a** (0.7 g, 38%). [α]_D_^20^ = +21.7 (*c* 1.0, CHCl_3_); FAB MS: m/z = 314.0 [M-OCH_2_CCH]^+^. Anal. Calcd for C_15_H_19_N_3_ O_8_ (369.3): C 48.78, H 5.19. Found: C 48.45, H 5.19.

B. A solution of **3a’’** (1.0 g, 2.46 mmol) and NaN_3_ 0.21 g, 3.2 mmol) in dry DMF (75 ml) was heated to 65 °C for 48 h until TLC indicated complete consumption of the starting material. Workup as described under A. and column chromatography (n-hexane/ethyl actate 1:1) afforded **4a** (0.42 g, 44%).

**4a’**

**2-Propynyl 6-azido-2,3,4-tri-*O*-benzoyl-6-deoxy-β-D-glucopyranoside (4a’)**

A. A solution of **3a’** (3.42 g, 5.0 mmol) and NaN_3_ (4.0 g, 60.0 mmol) in DMF/H_2_O (9:1, 55 ml) was heated to 80 °C for 48 h until TLC indicated complete consumption of the starting material. The solution was poured onto crushed ice and the mixture was extracted with dichloromethane (3 x 50 ml). The combined organic extracts were dried over Na_2_SO_4_, filtered and concentrated in vacuo. Column chromatography (n-hexane/ethyl actate 1/1) of the residue afforded **4a** (2.34 g, 84%). [α]_D_^20^ = +6.2 (*c* 1.0, CHCl_3_); FAB MS: m/z = 550 [M]^+^. Anal. Calcd for C_30_H_25_N_3_O_8_ (555.5): C 64.86, H 4.54. Found: C 65.11, H 4.80.

**2b**

**3-Butynyl 2,3,4-tri-*O*-acetyl-6-*O*-*p*-tolylsulfonyl-β-D-glucopyranoside (2b)**

Deacetylation of 3-butynyl 2,3,4,6-tetra-*O*-acetyl-β-D-glucopyranoside (**1b**) [3] (5.0 g, 12.5 mmol) as described for the preparation of **2a** afforded 3-butynyl β-D-glucopyranoside (2.86 g, 99%). [α]_D_^20^ = -33.9 (*c* 1.0, MeOH); FAB MS: m/z = 255.1 [M+Na]^+^. Tosylation of the latter (2.18 g, 9.39 mmol) as described for the preparation of **2a** afforded **2b** (3.29 g, 91%). [α]_D_^20^ = -25.2 (*c* 1.0, CHCl_3_); FAB MS: m/z = 409.0 [M+Na]^+^.

**3b**

**3-Butynyl 2,3,4-tri-*O*-acetyl-6-*O*-*p*-tolylsulfonyl-β-D-glucopyranoside (3b)**

Acetylation of **2b** (3.13g, 8.26 mmol) as described for the preparation of **3a** afforded **3b** (3.99 g, 94%) which was used for the next step without further purification. FAB MS: m/z = 443.1 [M-O(CH_2_)_2_CCH]^+^.

**4b**

**3-Butynyl 2,3,4-tri-*O*-acetyl-6-azido-6-deoxy-β-D-glucopyranoside (4b)**

Treatment of **3b** (1.0 g, 1.95 mmol) with NaN_3_ (1.26 g, 19.53 mmol) in DMF/H_2_O (9:1, 100 ml) as described for the preparation of **4a** under A. afforded **4b** (0.56 g, 74%). [α]_D_^20^ = -25.3 (*c* 1.0, CHCl_3_); FAB MS: m/z = 767.3 [M+H]^+^, 314.1 [M-O(CH_2_)_2_CCH]^+^.

**2c**

**2-Propynyl 6-*O*-*p*-tolylsulfonyl-α-D-glucopyranoside (2c)**

Deacetylation of 2-propynyl 2,3,4,6-tetra-*O*-acetyl-α-D-glucopyranoside (**1c**) (4.0 g, 7.8 mmol) as described for the preparation of **2a** afforded 2-propynyl α-D-glucopyranoside (1.68 g, 99%). [α]_D_^20^ = +130.2 (*c* 1.0, MeOH); FAB MS: m/z = 241.1 [M+Na]^+^. Tosylation of the latter (2.0 g, 9.17 mmol) as described for the preparation of **2a** afforded **2c** (1,45 g, 42%). [α]_D_^20^ = +93.4 (*c* 1.0, CHCl_3_); FAB MS: m/z = 395.0 [M+Na]^+^.

**3c**

**2-Propynyl 2,3,4-tri-*O*-acetyl-6-*O*-*p*-tolylsulfonyl-α-D-glucopyranoside (3c)**

Acetylation of **2c** (1.45g, 3.9 mmol) as described for the preparation of **3a** afforded **3c** (1.88 g, 96%) which was used for the next step without further purification. FAB MS: m/z = 499.1 [M+H]^+^, 443.0 [M-OCH_2_CCH]^+^.

**4c**

**2-Propynyl 2,3,4-tri-*O*-acetyl-6-azido-6-deoxy-α-D-glucopyranoside (4c)**

Treatment of **3c** (1.0 g, 2.0 mmol) with NaN_3_ (1.3 g, 20.0 mmol) in DMF/H_2_O (9:1, 75 ml) as described for the preparation of **4a** under A. afforded **4c** (0.52 g, 70%). [α]_D_^20^ = +151.8 (*c* 1.0, CHCl_3_); FAB MS: m/z = 314.2 [M-OCH_2_CCH]^+^.

**2d**

**2-Propynyl 6-*O*-*p*-tolylsulfonyl-β-D-galactopyranoside (2d)**

Deacetylation of 2-propynyl 2,3,4,6-tetra-*O*-acetyl-β-D-galactopyranoside (**1d**) [1] (5.0 g, 12.95 mmol) as described for the preparation of **2a** afforded 2-propynyl β-D-galactopyranoside (2.79 g, 99%). Tosylation of the latter (5.0 g, 22.94 mmol) as described for the preparation of **2a** afforded **2d** (5.8 g, 68%). [α]_D_^20^ = -55.5 (*c* 1.0, CHCl_3_); FAB MS: m/z = 373.2 [M+H]^+^.

**3d**

**2-Propynyl 2,3,4-tri-*O*-acetyl-6-*O*-*p*-tolylsulfonyl-β-D-galactopyranoside (3d)**

Acetylation of **2d** (2.5g, 6.72 mmol) as described for the preparation of **3a** afforded **3d** (2.98 g, 89%) which was used for the next step without further purification. FAB MS: m/z = 521.2 [M+Na]^+^.

**4d**

**2-Propynyl 2,3,4-tri-*O*-acetyl-6-azido-6-deoxy-β-D-galactopyranoside (4d)**

Treatment of **3d** (1.0 g, 2.0 mmol) with NaN_3_ (1.3 g, 20.0 mmol) in DMF/H_2_O (9:1, 100 ml) as described for the preparation of **4a** under A. afforded **4d** (0.25 g, 33%). [α]_D_^20^ = -32.5 (*c* 1.0, CHCl_3_); FAB MS: m/z = 314.1 [M-OCH_2_CCH]^+^. Anal. Calcd. for C_15_H_19_N_3_O_8_ (369.3): C, 48.78; H, 5.19; N, 11.38. Found: C, 49.10; H, 5.25; N, 11.24.

**2e**

**2-Propynyl 6-*O*-*p*-tolylsulfonyl-α-D-galactopyranoside (2e)**

Deacetylation of 2-propynyl 2,3,4,6-tetra-*O*-acetyl-α-D-galactopyranoside (**1e**) (2.56 g, 6.63 mmol) as described for the preparation of **2a** afforded 2-propynyl α-D-galactopyranoside (1.43 g, 99%). Tosylation of the latter (1.28 g, 5.87 mmol) as described for the preparation of **2a** afforded **2e** (1.0 g, 46%). [α]_D_^20^ = +110.1 (*c* 1.0, MeOH); FAB MS: m/z = 395.0 [M+Na]^+^.

**3e**

**2-Propynyl 2,3,4-tri-*O*-acetyl-6-*O*-*p*-tolylsulfonyl-α-D-galactopyranoside (3e)**

Acetylation of **2e** (0.67 g, 1.8 mmol) as described for the preparation of **3a** afforded **3e** (0.89 g, 99%) which was used for the next step without further purification. FAB MS: m/z = 443.0 [M-OCH_2_CCH]^+^.

**4e**

**2-Propynyl 2,3,4-tri-*O*-acetyl-6-azido-6-deoxy-α-D-galactopyranoside (4e)**

Treatment of **3e** (0.76 g, 1.52 mmol) with NaN_3_ (1.33 g, 20.4 mmol) in DMF/H_2_O (9:1, 50 ml) as described for the preparation of **4a** under A. afforded **4e** (0.22 g, 39%). [α]_D_^20^ = +98.5 (*c* 1.0, CHCl_3_); FAB MS: m/z = 370.3 [M+H]^+^.

**2f**

**2-Propynyl 6-*O*-*p*-tolylsulfonyl-α-D-mannopyranoside (2f)**

Deacetylation of 2-propynyl 2,3,4,6-tetra-*O*-acetyl-α-D-mannopyranoside (**1f**) [2,4] (5.0 g, 12.95 mmol) as described for the preparation of **2a** afforded 2-propynyl α-D-mannopyranoside (2.75 g, 98%). Tosylation of the latter (2.5 g, 11.46 mmol) as described for the preparation of **2a** afforded **2f** (2.7 g, 63%). [α]_D_^20^ = +71.7 (*c* 1.0, CHCl_3_); FAB MS: m/z = 385.0 [M+Na]^+^.

**3f**

**2-Propynyl 2,3,4-tri-*O*-acetyl-6-*O*-*p*-tolylsulfonyl-α-D-mannopyranoside (3f)**

Acetylation of **2f** (2.5 g, 6.7 mmol) as described for the preparation of **3a** afforded **3f** (2.1 g, 63%) which was used for the next step without further purification. FAB MS: m/z = 521.1 [M+Na]^+^.

**4f**

**2-Propynyl 2,3,4-tri-*O*-acetyl-6-azido-6-deoxy-α-D-mannopyranoside (4f)**

Treatment of **3f** (2.0 g, 4.0 mmol) with NaN_3_ (0.77 g, 12.0 mmol) in DMF/H_2_O (9:1, 50 ml) as described for the preparation of **4a** under A. afforded **4f** (1.23 g, 83%). [α]_D_^20^ = +63.4 (*c* 1.0, CHCl_3_); FAB MS: m/z = 370.0 [M+H]^+^.

**2g**

**3-Butynyl 6-*O*-*p*-tolylsulfonyl-α-D-mannopyranoside (2g)**

Deacetylation of 3-butynyl 2,3,4,6-tetra-*O*-acetyl-α-D-mannopyranoside (**1g**) [3] (5.0 g, 12.48 mmol) as described for the preparation of **2a** afforded 3-butynyl α-D-mannopyranoside (2.88 g, 99%). Tosylation of the latter (2.69 g, 11.59 mmol) as described for the preparation of **2a** afforded **2g** (2.58 g, 58%). [α]_D_^20^ = +32.7 (*c* 1.0, CHCl_3_); FAB MS: m/z = 409.1 [M+Na]^+^.

**3g**

**3-Butynyl 2,3,4-tri-*O*-acetyl-6-*O*-*p*-tolylsulfonyl-α-D-mannopyranoside (3g)**

Acetylation of **2g** (2.4 g, 6.22 mmol) as described for the preparation of **3a** afforded **3g** (2.62 g, 82%) which was used for the next step without further purification. FAB MS: m/z = 443.1 [M-O(CH_2_)_2_CCH]^+^.

**4g**

**3-Butynyl 2,3,4-tri-*O*-acetyl-6-azido-6-deoxy-α-D-mannopyranoside (4g)**

Treatment of **3g** (2.0 g, 3.9 mmol) with NaN_3_ (2.5 g, 39.0 mmol) in DMF/H_2_O (9:1, 150 ml) as described for the preparation of **4a** under A. afforded **4g** (1.01 g, 76%). [α]_D_^20^ = +58.4 (*c* 1.0, CHCl_3_); FAB MS: m/z = 384.1 [M+H]^+^.

**6**

***tert*-Butyl *N*-2-[(9*H*-Fluoren-9-ylmethoxy)carbonyl]-*N*-{[1-(2,3,4-tri-*O*-acetyl-6-deoxy-1-(2-propynyl)-β-D-glucopyranos-6-yl)-1*H*-1,2,3-triazol-4-yl]methyl}-L-α-asparaginate (6)**

To a solution of **4a** (221 mg, 0.6 mmol) and **5** (0.54 g, 1.2 mmol) in toluene (7.0 ml) was added under stirring at 0 °C diisopropyl ethyl amine (310 μl, 1.8 mmol) and (EtO)_3_PCuI (24 mg, 0.06 mmol). The mixture was warmed to room temperature and stirring was continued for 30 minutes until TLC indicated complete conversion of the starting material **4a**. Concentration of the mixture in vacuo and column chromatography of the residue (gradient ethyl acetate/n-hexane 1:1 to ethyl acetate) afforded **6** as amorphous white powder (0.3 g, 61%). [α]_D_^20^ = -9.5 (*c* 1.0, CHCl_3_); ESI MS: m/z = 840 [M+Na]^+^, 817 [M]^+^. Anal. Calcd for C_41_H_47_N_5_O_13_ x 5H_2_O (907.9): C 54.24, H 6.33, N 7.71. Found: C 54.25, H, 6.19, N 7.51.

A fraction also contained **7a** which, however, could not be obtained in pure form.

**7a**

**Bis-2,3,4-Tri-*O*-acetyl-6-deoxy-6-(4-hydroxymethyl-1H-1,2,3-triazol-1-yl)-β-D-glucopyranose 1,OH’:1’,OH-dianhydride (7a)**

A. A solution of **4a** (221 mg, 0.6 mmol), diisopropyl ethyl amine (310 μl, 1.8 mmol) and (EtO)_3_PCuI (24 mg, 0.06 mmol) in toluene (20 ml) was stirred at room temperature for 12 h until TLC indicated the complete consumption of the starting material. Concentration of the mixture in vacuo and column chromatography of the residue (gradient ethyl acetate/n-hexane 1:1 to ethyl acetate) afforded **7a** (120 mg, 54%) as a white amorphous solid. [α]_D_^20^ = +14.4 (*c* 1, CHCl_3_); ESI MS: m/z = 740 [M+H]^+^, 762 [M+Na]^+^; FAB MS: m/z = 739.2 [M+Na]^+^; HRFD MS: Calcd. m/z = 739.24171. [M+H]^+^. Found: m/z = 739.24161. Anal. Calcd for C_30_H_38_N_6_O_16_ x H_2_O (756,6): C 47.62, H 5.33, N 11.11. Found: C 48.01, H 5.60, N, 10.98.

B. Treatment of **4a** (221 mg, 0.6 mmol), diisopropyl ethyl amine (310 μl, 1.8 mmol) and (EtO)_3_PCuI (24 mg, 0.06 mmol) in toluene (20 ml) for 1 h at 80 °C under microwave irradiation (20 W) and workup as described under A. afforded **7a** (45 mg, 20%).

**8**

**Oligomer of 2-propynyl 6-azido-2,3,4-tri-*O*-benzoyl-6-deoxy-β-D-glucopyranoside (8)**

Treatment of **4a’** (333 mg, 0.6 mmol), diisopropyl ethyl amine (310 μl, 1.8 mmol) and (EtO)_3_PCuI (24 mg, 0.06 mmol) in toluene (20 ml) as described for the preparation of compound **7a** under A. afforded an inseparable mixture of oligomers **8** in various amounts.

FAB MS: m/z = 1111.6 [dimer+H]^+^, 1667.4 [trimer+H]^+^, 2223.7 [tetramer+H]^+^.

**7b**

**Bis-2,3,4-Tri-*O*-acetyl-6-deoxy-6-[4-(2-hydroxyethyl)-1H-1,2,3-triazol-1-yl]-β-D-glucopyranose 1,OH’:1’,OH-dianhydride (7b)**

Treatment of **4b** (230 mg, 0.6 mmol), diisopropyl ethyl amine (310 μl, 1.8 mmol) and (EtO)_3_PCuI (24 mg, 0.06 mmol) in toluene (20 ml) as described for the preparation of compound **7a** under B. afforded **7b** (74 mg, 32%). [α]_D_^20^ = +26.9 (*c* 1, CHCl_3_); HRFD MS: Calcd. m/z = 767.27301 [M+H]^+^. Found: m/z = 767.27250.

**7c**

**Bis-2,3,4-Tri-*O*-acetyl-6-deoxy-6-(4-hydroxymethyl-1H-1,2,3-triazol-1-yl)-α-D-glucopyranose 1,OH’:1’,OH-dianhydride (7c)**

A. Treatment of **4c** (221 mg, 0.6 mmol), diisopropyl ethyl amine (310 μl, 1.8 mmol) and (EtO)_3_PCuI (24 mg, 0.06 mmol) in toluene (20 ml) as described for the preparation of compound **7a** under A. afforded **7c** (30 mg, 14%). [α]_D_^20^ = -50.1 (*c* 1, CHCl_3_); HRFD MS: Calcd. m/z = 739.24171 [M+H]^+^. Found: m/z = 739.24130.

B. Treatment of **4c** (221 mg, 0.6 mmol), diisopropyl ethyl amine (310 μl, 1.8 mmol) and (EtO)_3_PCuI (24 mg, 0.06 mmol) in toluene (20 ml) as described for the preparation of compound **7a** under B. resulted in a complex mixture from which no pure **7c** could be isolated.

**7d**

**Bis-2,3,4-Tri-*O*-acetyl-6-deoxy-6-(4-hydroxymethyl-1H-1,2,3-triazol-1-yl)-β-D-galactopyranose 1,OH’:1’,OH-dianhydride (7d)**

Treatment of **4d** (221 mg, 0.6 mmol), diisopropyl ethyl amine (310 μl, 1.8 mmol) and (EtO)_3_PCuI (24 mg, 0.06 mmol) in toluene (20 ml) as described for the preparation of compound **7a** under A. afforded **7d** (62 mg, 28%). [α]_D_^20^ = -27.0 (*c* 1, CHCl_3_); HRFD MS: Calcd. m/z = 739.24171 [M+H]^+^. Found: m/z = 739.23983.

B. Treatment of **4d** (221 mg, 0.6 mmol), diisopropyl ethyl amine (310 μl, 1.8 mmol) and (EtO)_3_PCuI (24 mg, 0.06 mmol) in toluene (20 ml) as described for the preparation of compound **7a** under B. resulted in a complex mixture from which no pure **7d** could be isolated.

**7e**

**Bis-2,3,4-Tri-*O*-acetyl-6-deoxy-6-(4-hydroxymethyl-1H-1,2,3-triazol-1-yl)-α-D-galactopyranose 1,OH’:1’,OH-dianhydride (7e)**

Treatment of **4e** (221 mg, 0.6 mmol), diisopropyl ethyl amine (310 μl, 1.8 mmol) and (EtO)_3_PCuI (24 mg, 0.06 mmol) in toluene (20 ml) as described for the preparation of compound **7a** under A. and B. resulted in a complex reaction mixture from which no pure **7e** could be isolated.

**7f**

**Bis-2,3,4-Tri-*O*-acetyl-6-deoxy-6-(4-hydroxymethyl-1H-1,2,3-triazol-1-yl)-α-D-mannopyranose 1,OH’:1’,OH-dianhydride (7f)**

Treatment of **4f** (221 mg, 0.6 mmol), diisopropyl ethyl amine (310 μl, 1.8 mmol) and (EtO)_3_PCuI (24 mg, 0.06 mmol) in toluene (20 ml) as described for the preparation of compound **7a** under B. afforded **7f** (67 mg, 30%). [α]_D_^20^ = +35.4 (*c* 1, CHCl_3_); HRFD MS: Calcd. m/z = 739.24171. [M+H]^+^. Found: m/z = 739.24055.

**7g**

**Bis-2,3,4-Tri-*O*-acetyl-6-deoxy-6-[4-(2-hydroxyethyl)-1H-1,2,3-triazol-1-yl]-α-D-mannopyranose 1,OH’:1’,OH-dianhydride (7g)**

Treatment of **4g** (230 mg, 0.6 mmol), diisopropyl ethyl amine (310 μl, 1.8 mmol) and (EtO)_3_PCuI (24 mg, 0.06 mmol) in toluene (20 ml) as described for the preparation of compound **7a** under B. afforded **7g** (123 mg, 53%). [α]_D_^20^ = +29.4 (*c* 1, CHCl_3_); HRFD MS: Calcd. m/z = 767.27301 [M+H]^+^. Found: m/z = 767.27479.

**Table 1:** ^1^H-NMR data

| Cmpd. | H-1 (J_1,2_) | H-2 (J_2,3_) | H-3 (J_3,4_) | H-4 (J_4,5_) | H-5 (J_5,6a_) | H-6a (J_6a,6b_) | H-6b (J_5,6b_) | others |
| --- | --- | --- | --- | --- | --- | --- | --- | --- |
| **1c** | 5.29 d  (3.8 Hz) | 4.92 dd  (10.4 Hz) | 5.49 t  (9.9 Hz) | 5.09 t  (9.9 Hz) | 4.12-4.05 m  (4.0 Hz) | 4.28-4.25 m  (-) | 4.12-4.05 m  (2.2 Hz) | 4.28-4.25 m, OCH_2_; 2.46 t, CH;  2.10 s, 2.08 s, 2.03 s, CH_3_ |
| **1e** | 5.32 d  (3.5 Hz) | 5.17 dd  (10.9 Hz) | 5.36 dd  (9.4 Hz) | 5,47 br d  (-) | 4.28-4.24 m  (-) | 4.11 mc  (-) | 4.11 mc  (-) | 4.28-4.24 m, OCH_2_; 2.46 t, CH;  2.15 s, 2.09 s, 2.05 s (2 H), 1.99 s CH_3_ |
| **2a** | 4.36 d  (7.8 Hz) | 3.13-3.09 dd  (9.1 Hz) | 3.30 t  (9.0 Hz) | 3.21 t  (9.6 Hz) | 3.33-3.28 m  (5.7 Hz) | 4.09-4.05 m  (-10.8 Hz) | 4.25-4.19 m  (2.0 Hz) | 7.69 d, 7.32 d, 4 H, Ar^Tos^; 4.15-4.11 dd, 2 H, OCH_2_; 2.77 t, J=2.5 Hz, 1 H, CH; 2.34 s, 3 H, CH_3_. |
| **2b** | 4.29 d  (7.8 Hz) | 3.35 t  (-) | 3.52-3.44 m  (-) | 3.52-3.44 m  (-) | 3.52-3.44 m  (-) | 4.27 mc  (-) | 4.27 mc  (-) | 7.79 d, 7.33 d, Ar^Tos^; 3.81 m, 3.62 m, OCH_2_; 2.46-2.43 m, CH_2_; 2.46-2.43 m, CH_3_^Ar^; 2.03 t, CH |
| **2c** | 4.96 d  (3.81 Hz) | 3.35 dd  (-) | 3.72 d  (-) | 3.45 t  (-) | 3.77 m  (-) | 4.32-4.19 m  (-) | 4.32-4.19 m  (-) | 7.79 d, 7,33 d, Ar^Tos^; 4.32-4.19 m, OCH_2_;  2.48 t, CH; 2.43 s, CH_3_ |
| **2d** | 4.38-4.16 m  (-) | 3.61 mc  (-) | 3.61 mc  (-) | 3.92 br s  (-) | 3.75 t  (-) | 4.38-4.16 m  (-) | 4.38-4.16 m  (-) | 7.78 d, 7.33 d, Ar^Tos^; 4.38-4.16 m, OCH_2_;  2.52 t, CH; 2.42 s, CH_3_ |
| **2e** | 4.73 d  (3.6 Hz) | 3.44 t  (-) | 3.45 dd  (-) | 3.61 t  (-) | 3.72 dd  (-) | 4.15-3.97 m  (-) | 4.15-3.97 m  (-) | 7.77 d, 7.47 d, Ar^Tos^; 4.15-3.97 m, OCH_2_;  2.47 t, CH; 2.04 s, CH_3_ |
| **2f** | 4.96 s  (-) | 3.95 s  (-) | 3.79-3.75 m  (-) | 3.79-3.75 m  (-) | 3.79-3.75 m  (-) | 4.36-4.26 m  (-) | 4.36-4.26 m  (-) | 7.79 d, 7.33 d, Ar^Tos^;4.14 t, OCH_2_; 2.44 d, CH;  2.42 s, CH_3_ |
| **2g** | 4.81 s  (-) | 3.94 s  (-) | 3.82-3.78 m  (-) | 3.82-3.78 m  (-) | 3.82-3.78 m  (-) | 4.28 d  (-) | 4.35 dd  (-) | 7.80 d, 7.33 d, Ar^Tos^; 3.70-3.64 m,  3.54-3.47 m, OCH_2_; 2.42-2.39 m, CH_2_;  2.42-2.39 m, CH_3_; 1.97 t, CH |
| **3a** | 4.69 d  (8.0 Hz) | 4,94–4,88 m  (9.8 Hz) | 5.18 t  (9.4 Hz) | 4,94–4,88 m  (2.7 Hz) | 3.78-3.73 m  (3.1 Hz) | 4.14-4.10 m  (-11.1 Hz) | 4.09-4.05 m  (5.8 Hz) | 7.77 d, 7.34 d, 4 H, Ar^Tos^; 4.30-4.21 m, 2 H, OCH_2_; 2.46-2.45 t, J=2.3 Hz, 1 H, CH; 2.44 s,  3 H, CH_3_^Ar^; 2.03 s, 1.99 s, 1.98 s, 9 H, CH_3_. |
| **3a’** | 5.05 d  (8.0 Hz) | 5.46-5.42 dd  (9.6 Hz) | 5.85 t  (9.3 Hz) | 5.38 t  (9.8 Hz) | 4.09-4.06 m  (2.8 Hz) | 4.27-4.24 dd  (-11.2 Hz) | 4.21-4.17 dd  (6.3 Hz) | 7.94 d, 7.85 d, 7.79 d, 6 H, Ar^Bz^; 7.73 d, 2 H Ar^Tos^; 7.55-747 m, 3 H, Ar^Bz^; 7.43-7.33 m, 5 H, Ar^Bz^, Ar^Tos^; 7.28-7.22 m, 3 H, Ar^Bz^; 4.39-4.30 m, 2 H, OCH_2_; 2.43 t, J=2.5 Hz, 1 H, CH; 2.37 s,  3 H, CH_3_^Ar^. |
| **3a’’** | 4.81 d | 5.03-4.79 m | 5.52 t | 5.03-4.79 m | 3.77-3.72 m | 3.48 dd | 3.39 dd | 4.41 dd, OCH_2_; 2.55 t, CH;  2.06 s, 2.01 s, CH_3_ |
| **3b** | 4.50 d  (7.8 Hz) | 4.93-4.88 m  (9.6 Hz) | 5.17 t  (9.4 Hz) | 4.93-4.88 m  (1.7 Hz) | 3.74 mc  (-) | 4.13-4.04 m  (-10.9 Hz) | 4.13-4.04 m  (2.8 Hz) | 7.78 d, 7.36 d, Ar^Tos^; 3.83 -3.60 m, OCH_2_;  2.46 mc, CH_3_^Ar^; 2.42 m, CH_2_;  2.03 s, 2.00 s, 1.98 s, CH_3_; 1.96 t, CH |
| **3c** | 5.18 d  (3.8 Hz) | 4.79 dd  (10.17 Hz) | 5.43 t  (9.6 Hz) | 4.94 t  (9.6 Hz) | 4.11-4.06 m  (-) | 4.11-4.06 m  (-) | 4.11-4.06 m  (-) | 7.78 d, 7.35 d, Ar^Tos^; 4.20 t, OCH_2_; 2.45-2.43 m, CH_3_^Ar^, CH; 2.03 s, 2.00 s, 1.98 s, CH_3_ |

**Table 1:** Continuation

| **3d** | 4.76 d  (7.8 Hz) | 5.14 dd  (10.4 Hz) | 4.99 dd  (3.3 Hz) | 5.34 br d  (< 1.0) | 3.93 mc | 4.10 dd | 3.99 dd | 7.74 d, 7.33 d, Ar^Tos^; 4.29 s, OCH_2_; 2.46 s, CH;  2.43 s, CH_3_^Ar^; 2.03 s, 2.01 s, 1.94 s, CH_3_ |
| --- | --- | --- | --- | --- | --- | --- | --- | --- |
| **3e** | 5.26 d  (3.8 Hz) | 5.10 dd  (10.9 Hz) | 5.30 dd  (3.3 Hz) | 5.40 dd  (0.7 Hz) | 4.24 mc  (-) | 4.08 mc  (-) | 3.98 mc  (-) | 7.70 d, 7.35 d, Ar^Tos^; 4.20 t, OCH_2_; 2.45-2.43 m, CH_3_^Ar^, CH; 2.07 s, 2.05 s, 1.97 s CH_3_ |
| **3f** | 4.94 s  (-) | 5.22 dd  (3.5 Hz) | 5.29 dd  (9.9 Hz) | 5.16 t  (-) | 4.02 m  (-) | 4.12 mc | 4.12 mc | 7.79 d, 7.34 d, Ar^Tos^;4.20 d, OCH_2_; 2.47 d, CH, 2.45 s, CH_3_^Ar^; 2.12 s, 1.99 s, 1.97 s CH_3_ |
| **3g** | 4.77 d  (1.5 Hz) | 5.21 dd  (5.5 Hz) | 5.30 dd  (9.9 Hz) | 5.16 t  (-) | 4.13-4.06 m  (-) | 4.13-4.06 m  (-) | 4.13-4.06 m  (-) | 7.79 d, 7.35 d, Ar^Tos^; 3.75-3.56 m OCH_2_; 2.48-2.45 m, CH_3_^Ar^, CH_2_ ; 2.35 s CH;  2.12 s, 1.99 s, 1.97 CH_3_ |
| **4a** | 4.82 d  (8.1 Hz) | 5.01-4.96 m  (9.6 Hz) | 5.22 t  (9.3 Hz) | 4.95 t  (9.9 Hz) | 3.73-3.68 dq  (7.6 Hz) | 3.42-3.36 dd  (13.3 Hz) | 3.20-3.16 dd  (2.1 Hz) | 4.37-4.36 d, 2 H, OCH_2_; 2.47 t, J=2.1 Hz, 1 H, CH; 2.03 s, 2.01 s, 1.98 s, 9 H, CH_3_. |
| **4a’** | 5.17 d  (7.8 Hz) | 5.57-5.52 m  (9.5 Hz) | 5.92 t  (9.5 Hz) | 5.47 t  (9.8 Hz) | 4.06-4.01 dq  (7.5 Hz) | 3.60-3.55 dd  (-13.6 Hz) | 3.35-3.31 dd  (2.2 Hz) | 7.96 d, 7.91 d, 7.82 d, 6 H, 7.53-7.47 m, 2 H, 7.42-7.34 m, 5 H, 7.28-7.24 m, 2 H, Ar^Bz^; 4.52-4.38 m, 2 H, OCH_2_; 2.46 t, J=2.3 Hz, 1 H, CH. |
| **4b** | 4.61 d  (7.8 Hz) | 5.02-4.95 m  (-) | 5.21 t  (-) | 5.02-4.95 m  (2.5 Hz) | 3.72-3.64 m  (-) | 3.41  (-13.2 Hz) | 3.21  (7.3 Hz) | 3.97 m, 3.72-3.64 m OCH_2_; 2.48 m CH _2_; 2.05 s, 2.03 s, 2.01 s CH_3_; 1.96 t CH |
| **4c** | 5.31 d  (3.8 Hz) | 4.90 dd  (10.1 Hz) | 5.48 t  (9.4 Hz) | 5.02 t  (-) | 4.03 mc  (-) | 3.22 mc  (-) | 3.22 mc  (-) | 4.31 d, OCH_2_; 2.46 t, CH;  2.08 s, 2.04 s, 2.01 s, CH_3_ |
| **4d** | 4.79 d  (7.8 Hz) | 5.23 dd  (10.4 Hz) | 5.06 dd  (3.3 Hz) | 5.35 d  (-) | 3.59 mc  (-) | 3.59 mc  (-12.9 Hz) | 3.59 mc  (4.3 Hz) | 4.41 d, OCH_2_; 2.50 s, CH;  2.17 s, 2.08 s, 1.99s, CH_3_ |
| **4e** | 5.37-5.33 m  (-) | 5.17 dd  (-) | 5.37-5.33 m  (-) | 5.42 d  (-) | 4.18 mc  (-) | 3.16 dd  (-) | 3.45 dd  (-) | 4.31 t, OCH_2_; 2.47 t, CH;  2.16 s, 2.09 s, 1.99 s, CH_3_ |
| **4f** | 5.04 s  (-) | 5.28-5.23 m  (-) | 5.33 dd  (10.1 Hz) | 5.28-5.23 m  (-) | 3.98 mc  (-) | 3.35 m  (-) | 3.35 m  (-) | 4.31 d, OCH_2_; 2.52 t, CH;  2.17 s, 2.05 s, 1.99 s CH_3_ |
| **4g** | 4.87 d  (1.28 Hz) | 5.27-5.22 m  (-) | 5.34 dd  (-) | 5.27-5.22 m  (-) | 4.07-4.02 m  (-) | 3.39-3.28 m  (-) | 3.39-3.28 m  (-) | 3.97-3.81 m, 3.68-3.62m OCH_2_;  3.39-3.28 m; CH _2_; 2.16 s, 2.05 s, 1.99 s, CH_3_; 2.01 t, CH |
| **6** | 4.63-4.36 m  not resolved  (-) | 4.96 t  (9.1 Hz) | 5.21 t  (9.3 Hz) | 4.86 t  (9.6 Hz) | 3.82 t  (-) | 4.63-4.36 m  (-) | 4.27-4.18 m  (-) | 7.75 d, 7.56 d, 7.39 t, 7.29 t, 8 H, Ar^Fmoc^; 7.66 s, 1 H, Ar^Triazol^; 7.07 s, 5.90 d, NHCO; 4.63-4.36 m, 7 H, H-6_a_, CH_2_^Fmoc^, CH^Asp^, CH_2_^Triazol^, H-1; 4.27-4.18 m, 4 H, H-6_b_, CH^Fmoc^, C*H_2_*CC; 2.93-2.88 dd, 1 H, H^Asp-2a^; 2.62-2.57 dd, 1 H, H^Asp-2b^; 2.48 s, 1 H, CC*H*; 2.03 s, 2.02 s, 1.99 s, 9 H, CH_3_; 1.42 s, 9 H, CH_3_*^t^*^Bu^. |
| **7a** | 4.57 d  (8.0 Hz) | 5.00 dd  (9.5 Hz) | 5.23 t  (9.5 Hz) | 4.87 t  (9.5 Hz) | 4.13-4.08 m  (6.9 HZ) | 4.56 dd  (-14.2 Hz) | 4.34 dd  (8.9 Hz) | 5.04 d, 4.48 d, J=-13.5 Hz, OCH_2_;  7.35 s, H^Ar^; 2.12 s, 2.06 s, 2.01 s, CH_3_ |

**Table 1:** Continuation

| **7b** | 4.35 d  (8.1 Hz) | 4.99 br t  (9.4 Hz) | 5.24 t  (9.5 Hz) | 4.99 br t  (9.4 Hz) | 3.89-3.84 m  (1.6 Hz) | 4.67 dd  (-14.0 Hz) | 4.21 dd  (10.5 Hz) | 3.59-3.56 m, OCH_2_; 3.02-2.90 m, CH_2_Ar;  7.46 s, H^Ar^; 2.13 s, 2.06 s, 2.03 s, CH_3_ |
| --- | --- | --- | --- | --- | --- | --- | --- | --- |
| **7c** | 4.86 br s  (< 1.0 Hz) | 5.20 br d  (3.1 Hz) | 5.34 dd  (10.0 Hz) | 5.17 br t  (10.0 Hz) | 4.32-4.24 m  (-) | 4.58-4.46 m  (-) | 4.58-4.46 m  (-) | 4.32-4.24 m, OCH_2_;  7.74 s, H^Ar^; 2.14 s, 2.13 s, 1.99 s, CH_3_ |
| **7d** | 4.55 d  (9.5 Hz) | 5.22 dd  (10.4 Hz) | 5.04 dd  (3.3 Hz) | 5.56 br d  (< 1.0 Hz) | 4.29 br d  (8.5 Hz) | 4.54-4.52 m  (-14.1 Hz) | 4.41 dd  (8.4 Hz) | 5.06 d, 4.55 d, J=-13.5 Hz, OCH_2_;  7.35 s, H^Ar^; 2.13 s, 2.08 s, 1.99 s, CH_3_ |
| **7f** | 4.87 br s  (< 1.0 Hz) | 5.22 br d  (3.0 Hz) | 5.34 dd  (10.1 Hz) | 5.20 br t  (10.0 Hz) | 4.32-4.24 m  (-) | 4.85-4.46 m  (-) | 4.85-4.46 m  (-) | 4.32-4.24 m, OCH_2_;  7.74 s, H^Ar^; 2.14 s, 2.13 s, 1.99 s, CH_3_ |
| **7g** | 4.78 br s  (1.5 Hz) | 5.28 dd  (3.4 Hz) | 5.35 dd  (10.0 Hz) | 5.22 t  (10.0 Hz) | 4.14-6.07 m  (1.7 Hz) | 4.61 dd  (-14.0 Hz) | 4.33 dd  (10.4 Hz) | 3,22 dd, 3.09 dd, J=-16.0 Hz; OCH_2_; 2.95-2.90 m, CH_2_Ar; 7.42 s, H^Ar^; 2.18 s, 2.15 s, 2.92 s, CH_3_ |

**Table 2:** ^13^C-NMR data

| Cmpd. | C-1 | C-2 | C-3 | C-4 | C-5 | C-6 | OCH_2_ | CH_2_ | -C≡ | ≡CH | C-4^Ar^ | C-5^Ar^ | CH_3_^Ar^ | C=O | CH_3_ |
| --- | --- | --- | --- | --- | --- | --- | --- | --- | --- | --- | --- | --- | --- | --- | --- |
| **1c** | 94.5 | 70.4 | 69.8 | 68.3 | 67.8 | 61.6 | 55.4 | - | 78.1 | 75.3 | - | - | - | 170.6, 170.1, 170.0, 169.7 | 20.7, 20.6 |
| **1e** | 94.9 | 67.3 | 67.7 | 67.9 | 66.7 | 61.4 | 55.2 | - | 78.2 | 75.2 | - | - | - | 170.4, 170.3, 170.1, 169.9 | 20.7, 20.6 |
| **2a** | 101.9 | 70.9 | 74.5 | 77.6 | 74.9 | 70.6 | 56.4 | - | 79.7 | 76.4 | - | - | 21.6 | - | - |
| **2b** | 102.5 | 69.4 | 73.1 | 75.9 | 73.3 | 69.1 | 67.8 | 19.8 | 81.0 | 69.9 | - | - | 21.6 | - | - |
| **2c** | 97.1 | 74.1 | 73.9 | 69.4 | 69.9 | 69.0 | 54.9 | - | 78.6 | 75.4 | - | - | - | - | - |
| **2d** | 100.8 | 70.6 | 72.8 | 68.2 | 72.3 | 68.6 | 56.1 | - | 78.7 | 75.7 | - | - | 21.6 | - | - |
| **2e** | 97.2 | 68.5 | 68.6 | 67.5 | 68.7 | 70.5 | 53.6 | - | 79.5 | 77.3 | - | - | - | - | - |
| **2f** | 98.4 | 70.3 | 71.4 | 70.4 | 66.9 | 69.3 | 54.5 | - | 78.6 | 75.1 | - | - | 21.6 | - | - |
| **2g** | 99.8 | 70.2 | 71.4 | 70.4 | 66.9 | 69.4 | 65.7 | 19.6 | 81.8 | 69.7 | - | - | 21.6 | - | - |
| **3a** | 98.0 | 70.8 | 72.5 | 68.6 | 71.7 | 67.6 | 56.0 | - | 78.0 | 75.7 | - | - | 21.8 | 169.4, 170.3 | 20.6, 20.7 |
| **3a’** | 98.3 | 71.3 | 72.7 | 69.2 | 72.3 | 68.1 | 56.1 | - | 78.0 | 75.8 | - | - | 21.7 | 165.1, 165.5, 165.7 | - |
| **3a’’** | 97.9 | 70.9 | 72.5 | 70.9 | 73.2 | 30.6 | 55.9 | - | 78.0 | 75.7 | - | - | - | 170.2, 169.5, 169.4 | 20.6 |
| **3b** | 100.6 | 70.9 | 72.4 | 68.6 | 71.6 | 67.7 | 67.8 | 19.8 | 80.4 | 69.5 | - | - | 21.6 | 170.2, 169.4, 169.2 | 20.6, 20.5 |
| **3c** | 94.2 | 70.2 | 69.6 | 68.5 | 67.6 | 67.4 | 55.4 | - | 78.0 | 75.3 | - | - | 21.6 | 170.0, 169.4 | 20.6, 20.5 |
| **3d** | 98.6 | 68.2 | 70.5 | 66.8 | 70.7 | 66.2 | 55.9 | - | 78.0 | 75.5 |  |  | 21.6 | 170.3, 169.5 | 20.7, 20.5,20.4 |
| **3e** | 94.8 | 67.5 | 67.1 | 67.7 | 66.7 | 66.7 | 55.4 | - | 78.1 | 75.2 |  |  | 21.6 | 170.3, 169.9, 169.8 | 20.7, 20.6, 20.5 |
| **3f** | 95.9 | 69.2 | 68.7 | 66.1 | 68.8 | 68.1 | 54.9 | - | 77.8 | 75.7 | - | - | 21.6 | 169.2, 169.8, 169.7 | 20.8, 20.6, |
| **3g** | 97.4 | 69.9 | 63.2 | 66.2 | 68.8 | 68.1 | 66.3 | 19.6 | 80.5 | 68.5 | - | - | 21.6 | 170.0, 169.8, 169.7 | 20.8, 20.6, |
| **4a** | 97.8 | 70.9 | 72.4 | 69.6 | 73.8 | 51.1 | 55.9 | - | 77.9 | 75.6 | - | - | - | 170.2, 169.5, 169.4 | 20.6 |
| **4a’** | 98.2 | 71.5 | 72.7 | 70.3 | 74.5 | 51.4 | 56.0 | - | 78.0 | 75.9 | - | - | - | 165.2, 165.5, 165.8 | - |

**Table 2:** Continuation

| **4b** | 100.6 | 71.0 | 72.4 | 69.4 | 73.6 | 51.1 | 67.8 | 19.8 | 80.6 | 69.4 | - | - | - | 170.2, 169.5, 169.3 | 20.6 |
| --- | --- | --- | --- | --- | --- | --- | --- | --- | --- | --- | --- | --- | --- | --- | --- |
| **4c** | 94.3 | 70.3 | 69.4 | 69.4 | 69.1 | 50.8 | 55.4 | - | 78.0 | 75.1 | - | - | - | 170.1, 170.0, 169.6 | 20.6 |
| **4d** | 98.5 | 68.4 | 70.7 | 68.4 | 67.9 | 50.5 | 55.9 | - | 77.4 | 73.1 | - | - | - | 170.2, 170.0, 169.5 | 20.7, 20.6, 20.5 |
| **4e** | 94.8 | 67.3 | 67.6 | 68.7 | 68.5 | 50.6 | 55.4 | - | 78.1 | 75.2 | - | - | - | 170.3, 170.1, 169.8 | 20.7, 20.6 |
| **4f** | 96.0 | 66.9 | 68.6 | 69.2 | 70.4 | 50.9 | 55.0 | - | 77.8 | 75.7 | - | - | - | 169.6, 169.8, | 20.8, 20.7, 20.6 |
| **4g** | 97.3 | 69.4 | 68.7 | 67.1 | 70.1 | 66.4 | 55.0 | 19.7 | 80.5 | 69.9 | - | - | - | 170.0, 169.8, | 20.8, 20.7, 20.6 |
| **6**^a^ | 98.4 | 70.9 | 72.4 | 69.9 | 72.6 | 60.4 | 56.6 | - | 78.0 | 76.2 | 141.4 | 125.1 | - | 169.4, 169.8, 170.1 | 20.6, 20.7 |
| **7a** | 101.4 | 71.1 | 72.3 | 69.8 | 72.2 | 50.7 | 65.0 | - | - | - | 144.9 | 125.1 | - | 170.0, 169.7, 169.4 | 20.7 |
| **7b** | 100.8 | 71.0 | 72.2 | 70.3 | 72.6 | 51.0 | 69.6 | 26.0 | - | - | 143.1 | 123.7 | - | 170.0, 169.4 | 20.6, 20.5 |
| **7c** | 93.2 | 69.8 | 67.4 | 69.4 | 70.2 | 50.0 | 59.8 | - | - | - | 142.4 | 125.2 | - | 170.4, 169.9, 169.5 | 20.6, 20.5, 20.4 |
| **7d** | 101.8 | 68.3 | 70.6 | 68.3 | 71.4 | 50.7 | 64.8 | - | - | - | 142.4 | 125.6 | - | 170.1, 169.9, 169.5 | 20.8, 20.6, 20.5 |
| **7f** | 96.1 | 69.3 | 68.5 | 67.7 | 69.2 | 51.0 | 59.8 | - | - | - | 142.8 | 124.4 | - | 170.1, 169.8, 169.7 | 20.8, 20.6 |
| **7g** | 97.0 | 69.3 | 68.6 | 67.5 | 69.7 | 50.9 | 66.3 | - | - | - | 142.9 | 122.1 | - | 170.3, 169.9, 169.6 | 20.8, 20.6 |

^a^28.1 (3C, C(*C*H_3_)_3_), 35.2 (CH_2_-N), 37.2 (*C*H_2_-CO), 47.2 (CH^Fmoc^), 51.0 (CH-N), 67.3 (CH_2_^Fmoc^), 82.0 (*C*(CH_3_)_3_), 120-1, 125.1, 127.2, 127.9 (C^Fmoc^), 156.9 (OC=O), 170.7 (COO*t*Bu), 171.2 (CONH).

**References**

1. Mereyala, H. B.; Gurrala, S. R. *Carbohydr. Res.* **1998,** *307,* 351–354. doi:[10.1016/S0008-6215(97)10104-5](http://dx.doi.org/10.1016/S0008-6215(97)10104-5)
2. Kaufman, R. J.; Sidhu, R. S. *J. Org. Chem.* **1982,** *47,* 4941–4947. doi:[10.1021/jo00146a023](http://dx.doi.org/10.1021/jo00146a023)
3. Tietze, L. F.; Bothe, U. *Chem.–Eur. J.* **1998,** *4,* 1179–1183. doi:[10.1002/(SICI)1521-3765(19980710)4:7<1179::AID-CHEM1179>3.0.CO;2-F](http://dx.doi.org/10.1002/(SICI)1521-3765(19980710)4:7<1179::AID-CHEM1179>3.0.CO;2-F)
4. Fernandez-Megia, E.; Correa, J.; Rodríguez-Meizoso, I.; Riguera, R. *Macromolecules* **2006,** *39,* 2113–2120. doi:[10.1021/ma052448w](http://dx.doi.org/10.1021/ma052448w)
